# Sunlight Assisted Photocatalytic Degradation of Ciprofloxacin in Water Using Fe Doped ZnO Nanoparticles for Potential Public Health Applications

**DOI:** 10.3390/ijerph15112440

**Published:** 2018-11-01

**Authors:** Sourav Das, Soumen Ghosh, Ananyo Jyoti Misra, Ashok J. Tamhankar, Amrita Mishra, Cecilia Stålsby Lundborg, Suraj K. Tripathy

**Affiliations:** 1 School of Biotechnology, Kalinga Institute of Industrial Technology (KIIT), Bhubaneswar 751024, India; sddassourav52@gmail.com (S.D.); soumen.cbel@gmail.com (S.G.); ananyomisra@gmail.com (A.J.M.); ejetee@gmail.com (A.J.T.); amritamishrabio@gmail.com (A.M.); 2 Department of Public Health Sciences, Karolinska Institutet, SE 17177 Stockholm, Sweden; cecilia.stalsby.lundborg@ki.se; 3 School of Chemical Technology, Kalinga Institute of Industrial Technology (KIIT), Bhubaneswar 751024, India

**Keywords:** antibiotic residues, aquatic environment, ciprofloxacin, Fe-doped ZnO nanoparticles, photocatalysis, sunlight

## Abstract

Antibiotic residues in the aquatic environment have the potential to induce resistance in environmental bacteria, which ultimately might get transferred to pathogens making treatment of diseases difficult and poses a serious threat to public health. If antibiotic residues in the environment could be eliminated or reduced, it could contribute to minimizing antibiotic resistance. Towards this objective, water containing ciprofloxacin was treated by sunlight-assisted photocatalysis using Fe- doped ZnO nanoparticles for assessing the degradation potential of this system. Parameters like pH, temperature, catalytic dosage were assessed for the optimum performance of the system. To evaluate degradation of ciprofloxacin, both spectrophotometric as well as microbiological (loss of antibiotic activity) methods were employed. 100 mg/L Fe-doped ZnO nanoparticle catalyst and sunlight intensity of 120,000–135,000 lux system gave optimum performance at pH 9 at 30 °C and 40 °C. Under these conditions spectrophotometric analysis showed complete degradation of ciprofloxacin (10 mg/L) at 210 min. Microbiological studies showed loss of antibacterial activity of the photocatalytically treated ciprofloxacin-containing water against *Staphylococcus aureus* (10^8^ CFU) in 60 min and for *Escherichia coli* (10^8^ CFU) in 75 min. The developed system, thus possess a potential for treatment of antibiotic contaminated waters for eliminating/reducing antibiotic residues from environment.

## 1. Introduction

Antibiotic residues in the environment is pose a major public health challenge [1]. Fluoroquinolones (FQs) are a class of environmentally stable broad spectrum antibiotics, which inhibits the enzymes DNA topoisomerase II (Gyrase) and DNA topoisomerase IV in bacteria thus interfering with their DNA replication machinery [2,3]. FQs are effective against both Gram positive and Gram negative bacteria and are used both in humans and animals. Ciprofloxacin is the most commonly used FQ, Studies report the occurrence of FQs, including ciprofloxacin, in water bodies worldwide [4]. FQ reaches water bodies through excretion after incomplete metabolism within the human/animal gut [5]. Their presence at up to 87 microgram/L and 31 mg/L has been demonstrated in wastewater discharge [6]. Conventional wastewater treatment including biological oxidation and other chemical and physical process leads to only partial removal of these compounds [7]. As a consequence, the presence of broad spectrum antibiotics like FQs, even at very minute concentrations, poses a threat to the surrounding ecosystem and human health through the development of antibiotic resistance amongst environmental bacteria [8], which can potentially lead to further spread of resistance to other bacterial populations including human and animal pathogens through processes such as ingestion of untreated or partially purified water or horizontal gene transfer [9].

**Figure 1:**
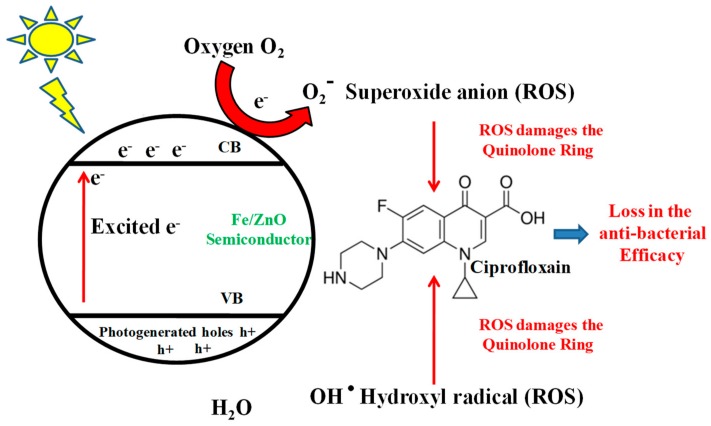
Schematic representation showing generation of reactive oxygen species (ROS) by Fe ZnO nanoparticles on activation with sunlight, and how these ROS attack active components of FQ to degrade them and reduce their anti-bacterial activity.

With the immediate necessity for substantive degradation of such organic environmental pollutants, semiconductor photocatalysis more appropriately, Advanced Oxidation Processes (AOPs) have proven quite useful [10]. They normally use a semiconductor metal oxide or one of its doped variants as a photo-oxidant which in presence of light charges up and leads to the generation of highly reactive oxidative species like hydroxyl radicals (OH·), superoxide anion (O_2_·−) and hydrogen peroxide (H_2_O_2_) for remediation of organic pollutants. The basic principle behind their action is shown in Figure 1. To date TiO_2_ and ZnO has been reported to be the best catalysts for photocatalytic applications because of their optical properties, thus having a much better quantum efficiency under visible light [11]. Moreover, owing to their high chemical stability, high oxidation efficiency, low toxicity, less cost, easy availability and being abundant in Nature they are excellent photocatalysts for the mineralization of organic pollutants in both acidic and basic media [12]. ZnO absorbs a substantial amount in the UV range [12] and UV accounts for only 3–5% of the sunlight, thus there is insufficient usage of the total sunlight available, so efforts are needed to design catalysts which will show better photocatalytic efficiency in the visible region of sunlight [11]. In order to address such problems, modifying the metal oxide semiconductor with transition, alkaline and rare earth metals like Mn, Fe, Co, Ni, Ag, Mg, Pb, N, C, S, P, is done [11], which will shift the light absorption towards the visible range.

Photocatalysis with ZnO for the degradation of antibiotics like ciprofloxacin, amoxicillin, ampicillin, cloxacillin using different sources of light was performed earlier [13,14]. Nearly 50% degradation of antibiotics was achieved with high rate constant and maximium degradation was reported at pH 10–11. It has been previously reported in one of our studies that using Fe-doped ZnO for photocatalytic applications majorly contributes towards the generation of H_2_O_2_ in the system, which ultimately is detrimental for the photocatalytic oxidation. Moreover the presence of Fe in the system, serves as an added advantage for the photocatalytic oxidation, since it comes in contact with H_2_O_2_ in the system to generate more of hydroxyl radicals via the Fenton process [15]. This will ultimately magnify the oxidation of antibiotic-containing water. Thus doping the catalyst with iron has some added benefits as far as increasing the photocatalytic efficiency of ZnO are concerned. Earlier such Fe-doped ZnO has been used for the successful degradation of wastewater containing dye molecules [16]. The aim of this study was to evaluate sunlight-assisted photocatalytic degradation of ciprofloxacin using Fe-doped ZnO nanoparticles. Further, the residual antibacterial activity of the treated water was assessed against a Gram positive (*Staphylococcus aureus*) and a Gram negative (*Escherichia coli*) bacterium.

## 2. Materials and Methods 

### 2.1. Materials

Chemicals used in this study include ciprofloxacin hydrochloride (MP Biomedicals, Santa Ana, CA, USA), zinc nitrate hexahydrate (98%, Sigma Aldrich, USA), trisodium citrate dihydrate (Sigma Aldrich, St. Louis, MO, USA), ferric chloride (Himedia, Mumbai, India), Luria agar and Luria broth (Himedia, Mumbai, India), sodium hydroxide (Merck, Kenilworth, NJ, USA), and hydrochloric acid (35.5%, Merck). All the chemicals were of molecular grade.

### 2.2. Preparation of Ciprofloxacin Stock Solution

Ciprofloxacin hydrochloride stock solution (100 mg L^−1^) was prepared in deionized water (NaOH was used to solubilize the ciprofloxacin followed by 5 min of ultrasonication), 2 L at a time and stored in dark at 4 °C. Working solutions of 10 mg L^−1^, (in 300 mL deionized water at a time) were prepared for each photocatalysis experiment, as required.

### 2.3. Synthesis of Nanocrystalline Fe-Doped ZnO

Fe-doped ZnO was prepared using a precipitation route as previously described [15]. Briefly, Zinc nitrate hexahydrate (5.948 g), ferric chloride (0.108 g) and trisodium citrate (5.882) were dissolved in 500 mL distilled water and stirred at 80 °C for 60 min. Then 250 mL of NaOH (250 mM) was slowly added dropwise into the solution using a burette until yellowish-white precipitate was formed. The precipitate was allowed to come to room temperature and was then centrifuged (10,000 rpm, which corresponds to 9391 g force, 10 min, Eppendorf 5424, USA), and rinsed with distilled water thrice. The precipitate was then dried at 70 °C overnight followed by calcination at 500 °C. The calcined Fe-doped ZnO powder was characterized as mentioned in our previous paper and used for photocatalytic applications.

### 2.4. Photocatalytic Degradation of Ciprofloxacin

A 300 mL aqueous solution of ciprofloxacin with a concentration of 10 mg L^−1^ was placed in a 500 mL borosilicate beaker with the required amount (see below) of Fe-doped ZnO and mixed by a magnetic stirrer. The mixture was kept undisturbed in dark for 30 min to allow equilibrium. The experiments were performed with different catalyst concentrations 100, 150 and 200 mg L^−1^, at pH 2, 3, 5.5, 7, 9, 10 and 11 (required pH was adjusted with 1 N HCl or 1 N NaOH), different reaction temperatures of 30 °C, 40 °C, 50 °C and 60 °C and different photocatalysts (TiO_2_ and ZnO) at a light intensity of 80,000 ± 3000 lux, which corresponds to 650 W/m^2^. At 15 min intervals, up to 210 min, collected samples were filtered through centrifugation (10,000 rpm, which corresponds to 9391 g force, 10 min, Eppendorf 5424) before spectrophotometric analysis (λ_max_-280 and 320 nm using a Shimadzu UV-1800 instrument (Japan) and the microbiology experiments for assessment of residual antibacterial activity. The time dependent decrease in absorbance values at λ_max_-280 and 320 nm suggests degradation of the antibiotic [14].

### 2.5. Residual Antibacterial Activity of the Treated Water

Qualitative assays were performed to assess the residual antibacterial activity of the treated water after photocatalytic degradation against the fully susceptible test organisms *Staphylococcus aureus* (MTCC code 3160) and *Escherichia coli* (MTCC code 7410) from the Microbial Type Culture Collection (MTCC, Chandigarh, India). The well diffusion method, according to the Clinical and Laboratory Standards Institute (CLSI) [17,18] was employed. All plates were prepared in 90 mm sterile Petri dishes (Tarsons, Mumbai, India) with 22 mL of Luria Bertani agar, yielding a depth of 4 mm. Test microorganism’s 100 µL of inoculum suspensions (OD_600_-0.5, corresponding to 1.0 × 10^8^ CFU mL^−1^) were poured into the agar plates when the temperature reached around 40–45 °C using a sterile micropipette, and homogenized thoroughly by mixing in a circular motion (pour-plate technique). After solidification, roundwells (6.0 mmin diameter) were punched into the seeded agar plates with a 6 mm cork borer. The wells were filled with 40 µL of the treated water samples (collected and filtered after regular time intervals) using a sterile micropipette. These plates were allowed to stand at 4 °C for 2 h and then incubated at 37 °C for 24 h. Three sets of simultaneous controls were used. One control was the organism control and consisted of a seeded Petri dish with no photocatalytically treated antibiotic sample. In the second control, samples were introduced in the holes of unseeded Petri dishes to check for sterility. Finally, to ensure the elimination of any solvent effect, wells filled with 40 µL of sterile double distilled water were run simultaneously as a third control. The diameters of the inhibition zones (zone of inhibition—ZOI) were measured in millimeters [19]. Each test was repeated six times and the mean values from the replicates along with standard error of mean (SEM) were calculated.

## 3. Results and Discussion

### 3.1. Photocataltytic Degradation of Ciprofloxacin and Process Optimization

**Figure 2:**
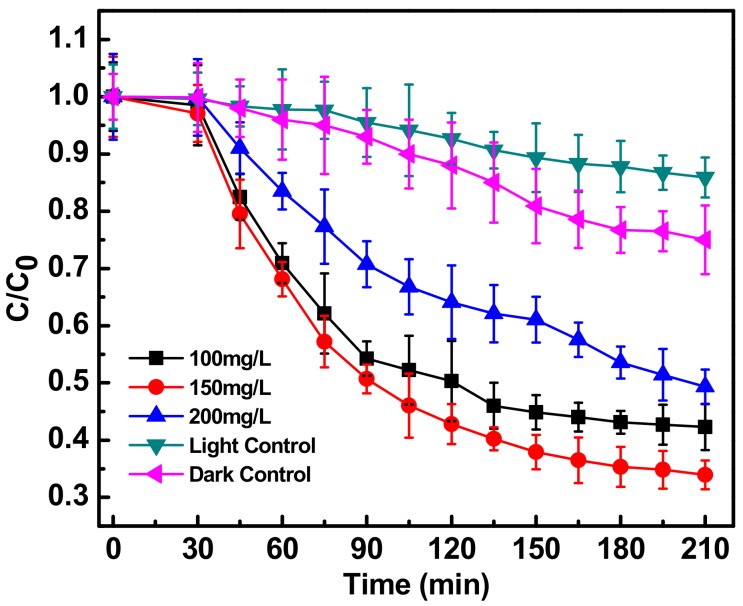
Photocatalytic degradation of antibiotic ciprofloxacin (10 mg/L) in water, in the presence of Fe-ZnO nanoparticles (at different concentrations of 100, 150 and 200 mg/L) irradiated with sunlight light intensity of 80,000 ± 3000 lux compared to photolysis (light control) and degradation in the absence of light (dark control). C_0_ represents initial concentration of ciprofloxacin and C represents concentration of ciprofloxacin at a particular time point. C/C_0_ denotes the time dependent change in ciprofloxacin concentration with respect to initial concentration.

Figure 2 shows the decrease in the C/Co absorption spectrum of ciprofloxacin (C = concentration at a particular time, Co = initial concentration of ciprofloxacin) at three catalyst concentrations (100, 150, 200 mg L^−1^), during sun-assisted photocatalysis by Fe-doped ZnO nanoparticles. The values were calculated on the basis of intensity of the absorbance peaks at 280 and 320 nm. At both these λ_max_, the absorbance showed a decreasing trend at all the three catalyst concentrations. 

A catalyst concentration of 150 mgL^−1^ caused a significant degradation of ciprofloxacin (10 mg L^−1^) of up to 66% in 210 min and was found to be optimum. The other two concentrations were not as effective. The 100 mg L^−1^ catalyst may not have the capability for substantial generation of reactive oxygen species, while the 200 mg L^−1^ catalyst concentration may be high enough to create a catalyst shielding effect. Moreover the 200 mg L^−1^ may possess slow or improper degradation kinetics of only 51%. For further experiments, therefore all the degradation experiments were carried out with 150 mg/L of Fe-doped ZnO. There was no significant change in concentration of the ciprofloxacin due to the direct sunlight assisted photolysis (light control) which was found to be only 14% [14]. The decrease in C/Co value (up to 25%) of the antibiotic when subjected to dark control reaction (at the optimum photocatalyst concentration of 150 mg/L), may be attributed to direct adsorption of the antibiotic in the presence of doped ZnO nanoparticles [11]. 

**Figure 3:**
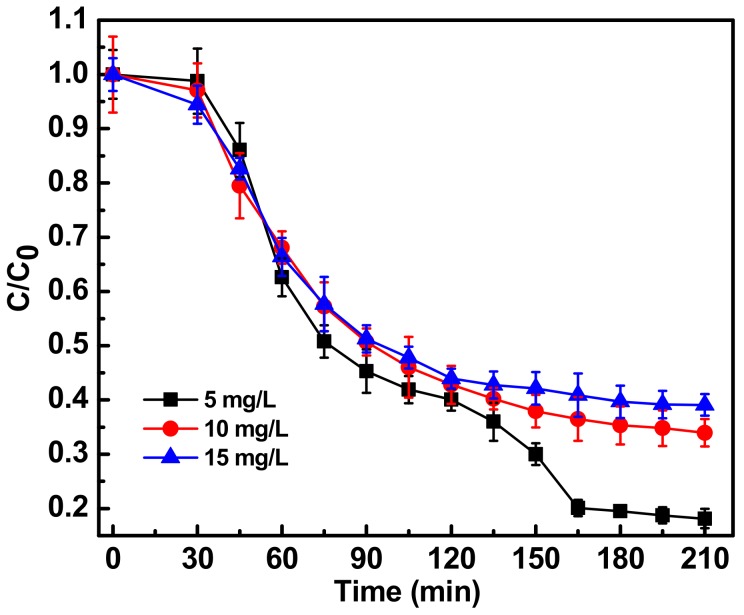
Photocatalytic degradation of antibiotic ciprofloxacin in water at different antibiotic concentration between 5, 10, 15 mg/L with optimum Fe-doped ZnO nanoparticles concentration of 150 mg/L and irradiated with sunlight intensity of 80,000 ± 3000 lux. C_0_ represents initial concentration of ciprofloxacin and C represents concentration of ciprofloxacin at a particular time point. C/C_0_ denotes, time dependent change in ciprofloxacin concentration with respect to initial concentration.

The concentration of antibiotic in the wastewater system is a key parameter to optimize the photocatalytic degradation process. A study was performed with ciprofloxacin concentrations of 5, 10 and 15 mg L^−1^. Figure 3 shows the photocatalytic degradation pattern of different concentrations of ciprofloxacin with the optimized concentration of Fe-doped ZnO nanoparticles. At 10 mg L^−1^ concentration no peaks were observed at 280 and 320 nm after 210 min of photocatalytic treatment, suggesting complete degradation of the quinolone ring. Five mg L^−1^ concentrations of ciprofloxacin were also completely degraded. Since studies with 10 mg L^−1^ concentrations were previously done and reported, the rest of the photocatalytic study were done with 10 mg L^−1^ concentration. With 15 mg L^−1^ ciprofloxacin concentration the degradation kinetics were a bit slower. Possible reasons could be a catalyst shielding effect and over-occupied catalyst active sites at 15 mg/L concentration [11,19].

**Figure 4:**
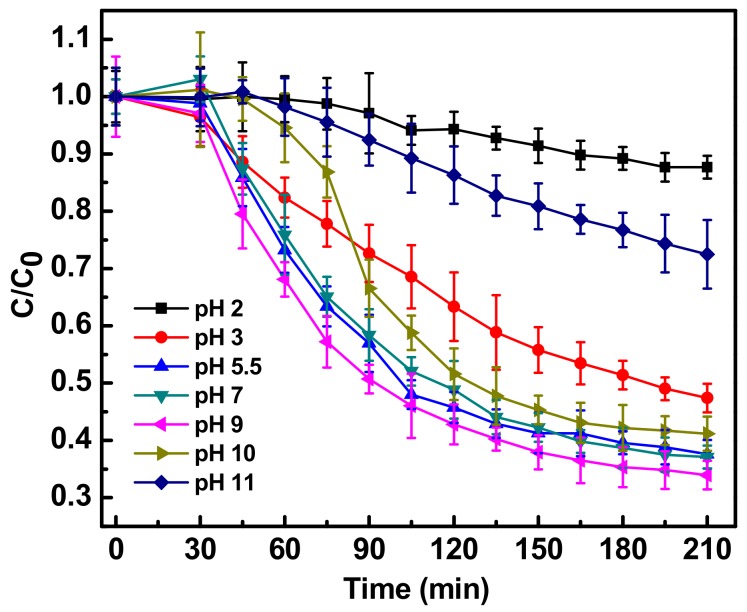
Photocatalytic degradation of antibiotic ciprofloxacin (10 mg/L) in water in the presence of Fe-ZnO nanoparticles (150 mg/L) irradiated with sunlight intensity of 80,000 ± 3000 lux at different reaction pH of 2, 3, 5.5, 7, 9, 10, 11. C_0_ represents initial concentration of ciprofloxacin and C represents concentration of ciprofloxacin at a particular time point. C/C_0_ denotes the time dependent change in ciprofloxacin concentration with respect to initial concentration.

pH modifies the surface charge properties of Fe-doped ZnO and possibly the chemical structure of the antibiotic, therefore the influence of pH on the photocatalytic activity of Fe-doped ZnO nanoparticles was studied by altering the pH of the reaction mixture in both the acidic and basic range. Figure 4 shows the effect on the photocatalytic degradation on ciprofloxacin of different pHs in the presence of Fe-doped ZnO nanoparticles. The best degradation efficiency of ciprofloxacin with Fe-doped ZnO nanoparticles, nearly 65%, was seen at pH 9, while the lowest degradation, only 10%, was observed at pH 2 [14]. The maximum ciprofloxacin degradation was thus obtained at basic pH values between 9 and 11 under solar light, where the available hydroxyl ions in the system can react with the valence band holes (h+) to form reactive hydroxyl radicals (OH·), which possesses high oxidation capability under photocatalytic conditions, subsequently enhancing the rate of photocatalytic degradation of ciprofloxacin. Similar results for the degradation of aromatic compounds were reported earlier [20]. At an acidic pH value of 2, the solar photocatalytic degradation of ciprofloxacin was hindered due to the high proton concentration, which possesses higher attraction for the hydroxyl anions, quenching the formation of hydroxyl radicals. As free hydroxyl ions in the system are decreased, the formation of hydroxyl radicals becomes limiting. Thus photocatalytic degradation of ciprofloxacin decreased at lower pH. It may also be possibly due to dissolution of Fe-doped ZnO under acidic conditions. Similar observations were previously made in the photocatalytic degradation of azo dyes [16].

Ciprofloxacin is an ampholytic compound with a pKa value of 6.09 for the carboxylic group and 8.74 for the nitrogen on the piperazinyl ring. The isoelectric or zwitterionic point is at pH 7.4. Thus ciprofloxacin seemed to be most sensitive to photocatalytic degradation at a pH closer to its zwitterionic form, i.e. at basic pH 9. It has earlier been reported that the maximum stability of the molecule was observed in reaction solution of pH 4.0 [21], where the carboxylic group is un-ionized and basic nitrogen is completely protonated. This adds an advantage to the ciprofloxacin pharmaceutically, because most of the pharmaceutical formulation possess pH between 3.5 and 5.5. This seems good from a pharmaceutical perspective but photocatalytic degradation at such low pH will be a challenge. Interestingly, it has been previously reported that, hospital wastewater flowing to drains has an pH in between 6.7 to 7.7 throughout the year, Moreover the pH of surface waters (mainly lakes and rivers) in India is between 6.5 to 8.5 [22]. The current study thus finds it application for degradation of antibiotics in hospital wastewater and surface water, since at this pH range the photocatalytic degradation was more than 60%, as shown in Figure 3.

From experimental observations and previous reports on the photocatalytic degradation of organic molecules like dyes [23] and antibiotics [24], we assumed that upon irradiation with solar light, within the Fe-doped ZnO nanoparticles, excitation of electrons takes place from the valence band into the conduction band. Photogenerated holes in the conduction band upon reacting with water molecules in the system generate hydroxyl radicals which possess oxidative nature and can get rid of antibiotics adsorbed on the Fe-doped ZnO surface. Moreover the high oxidative potential of valence band holes can also lead to the direct and indirect oxidation of antibiotics. The presence of Fe in the system possesses an added advantage to this photocatalytic degradation process. The presence of Fe delays the electron whole recombination, acting as one of the terminal acceptors of electrons, which eventually increases the generation of hydroxyl radicals and reactive species in the system. Also Fe as a Fenton agent is capable of producing reactive oxygen species like OH· radicals through the Fenton process, adding more ROS to the system for subsequent degradation of ciprofloxacin [11,25].

**Figure 5:**
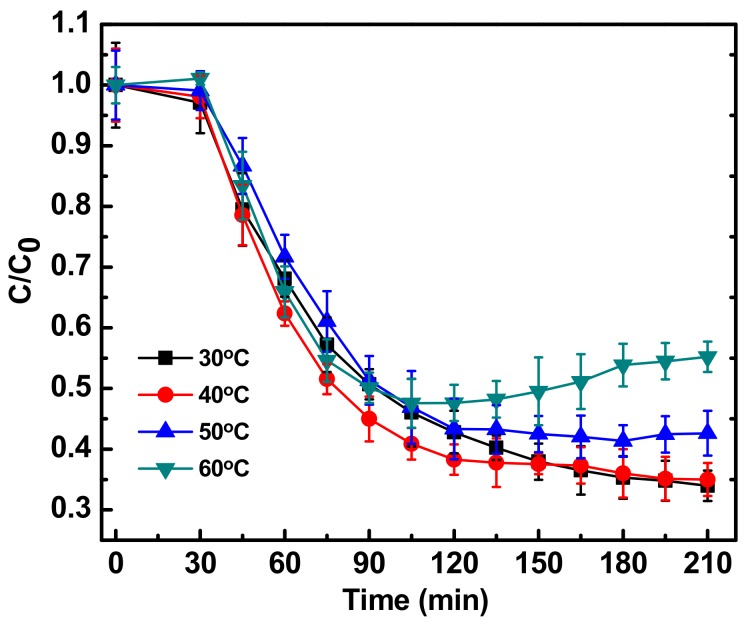
Photocatalytic degradation of antibiotic ciprofloxacin (10 mg/L) in water in the presence of Fe-ZnO nanoparticles (150 mg/L) irradiated with sunlight intensity of 80,000 ± 3000 lux and pH 9 with different reaction temperature. C_0_ represents initial concentration of ciprofloxacin and C represents concentration of ciprofloxacin at a particular time point. C/C_0_ denotes the time dependent change in ciprofloxacin concentration with respect to initial concentration.

Temperature was found to modulate the degradation kinetics (Figure 5). Generally it has been reported that with an increase in temperature the degradation kinetics are enhanced [11], but in the current study, the opposite trend was observed. With increasing temperature, the degradation kinetics decreased up to 60 °C. A possible reason could be the increase in the stability of fluoroquinolones on exposure to heat stress. It has been reported by Roca et. al. [26], that FQs can be stable at temperatures up to 120 °C. In a country like India, where the atmospheric temperature can reach up to 50 °C, the technique presented in this paper can be employed for successful degradation of ciprofloxacin and maybe other fluoroquinolones also, in wastewater matrices. The technique presented in this paper may also find its application for the treatment of hospital, pharmaceutical or industrial wastewater for the degradation of many organic molecules.

### 3.2. Analysis of Residual Antibacterial Activity of Antibiotic after Photocatalytic Degradation

Ciprofloxacin, as already discussed, is an antibiotic that belongs to the FQ class of antibiotics. The antibiotics that belong to this group, generally inhibit the growth of several microorganisms via the inhibition of DNA Gyrase, which is a factor is responsible for the division of bacterial cells. ciprofloxacin is active against a wide spectrum of Gram positive and Gram negative bacteria and ciprofloxacin and antibiotics of the FQ group are widely present in wastewaters such as those from hospital, municipal, pharmaceutical industry sources, etc. [1,22,27]. The residues of these antibiotics in the wastewaters generate antibiotic resistant bacteria in the environment, which is a potential major threat to public health.

**Figure 6:**
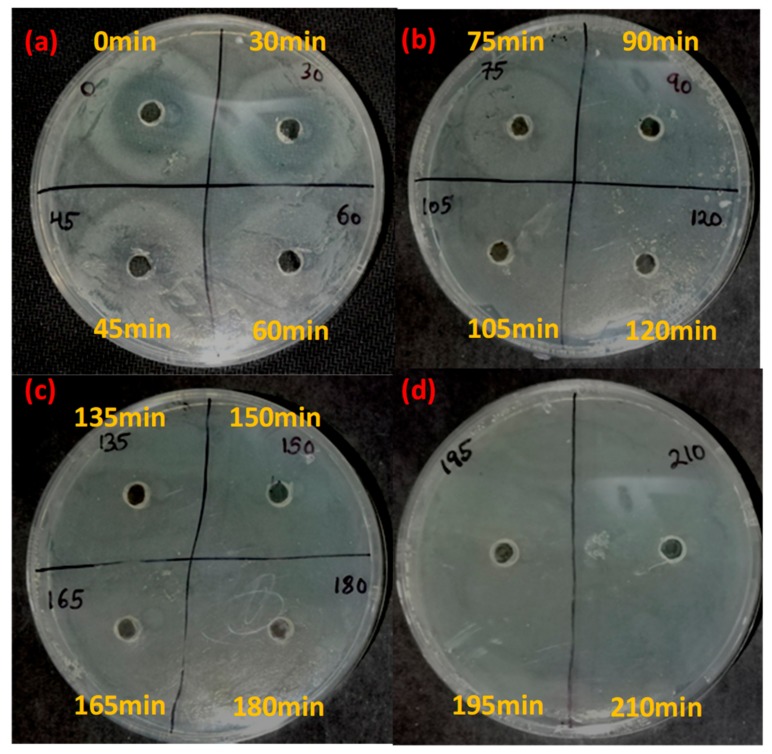
Residual antibiotic activity of the antibiotic ciprofloxacin after photocatalytic degradation with Fe-doped ZnO nanoparticles against *Staphylococcus aureus*. Yellow markings denote the time points at which sampling has been done. (**a**), (**b**), (**c**), (**d**) denotes the zone of inhibition shown by the antibiotic slurry collected at different time intervals.

**Figure 7:**
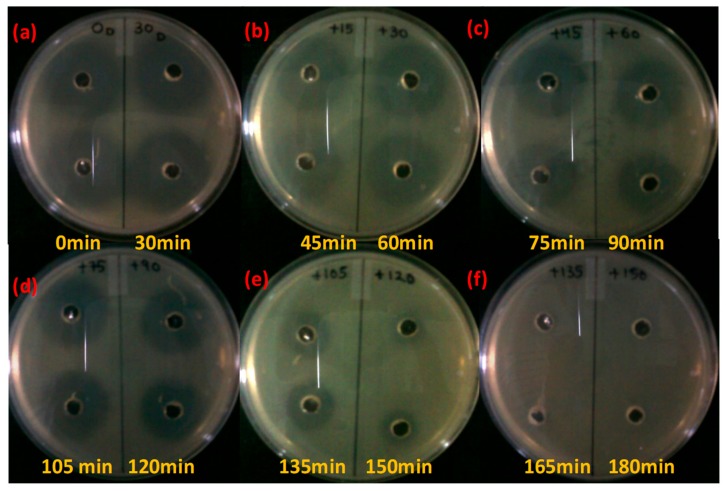
Residual antibiotic activity of the antibiotic ciprofloxacin after photocatalytic degradation with Fe Doped ZnO nanoparticles against *Escherichia coli*. Yellow marking denotes the time points at which sampling has been done. (**a**), (**b**), (**c**), (**d**), (**e**), (**f**) denotes the zone of inhibition shown by the antibiotic slurry collected at different time intervals.

The current work aims to employ photocatalysis for the successful degradation of the antibiotic ciprofloxacin. After subjecting ciprofloxacin to photocatalytic treatment with Fe-doped ZnO nanoparticles, a confirmatory bacterial inhibition experiment was conducted to check whether the antibiotic was completely degraded in the experimental system using as test organisms *Staphylococcus aureus* and *Escherichia coli* [19]. The results of the experiments (Table 1 and Figure 6 and Figure 7) showed that for both *Staphylococcus aureus* and *Escherichia coli*, ciprofloxacin lost its antibacterial activity after 60 minutes and 75 minutes post-irradiation, respectively. With increasing time, a decreasing zone of inhibition in both *Staphylococcus aureus* and *Escherichia coli* was evident. The zone of inhibition decreased from 12 mm to 5.5 mm and from 15 mm to 6 mm in the case of *Staphylococcus aureus* and *Escherichia coli* in 60 min and 75 min post-irradiation, respectively.

It can be seen that *Escherichia coli*, a Gram negative organism, shows susceptibility to ciprofloxacin that has been collected 75 minutes post-irradiation, which is slightly less than that of *Staphylococcus aureus* (sample collected 60 minutes post-irradiation), before completely showing zero susceptibility in both cases. As a Gram negative microorganism *Escherichia coli* has a weak cell wall that is made up of lipopolysaccharides [28,29]. Therefore it is easy for a disinfecting agent to penetrate its cellular defenses. compared to *Staphylococcus aureus*, which is Gram positive. In the case of the light control and dark control, antibacterial activity was not lost even after 120 min for both *Escherichia coli* and *Staphylococcus aureus*. There was little decrease in the zone of inhibition parameters and it clearly signified that ciprofloxacin was still present in the case of experimental controls, suggesting that both the photocatalyst (Fe-doped ZnO) and sunlight are indispensable in the degradation process.

## 4. Conclusions

An Fe-doped ZnO nanoparticles-based sunlight-assisted photocatalytic system was developed for the degradation of the fluoroquinolone antibiotic ciprofloxacin in water, assessing its best performance parameters like pH, temperature, and catalyst dosage. The degradation of ciprofloxacin was proved both spectrophotometrically as well as microbiologically by the loss of antibiotic activity of the photocatalytically treated water. The developed Fe-doped ZnO nanoparticles-based photocatalytic system can potentially be used for the degradation of other fluoroquinolones and other antibiotics as well as other organic contaminants in water. Antibiotic residues in aquatic systems have the potential to induce resistance in bacteria, which has further the potential to infect humans and thereby become a serious threat to human health. The developed system has therefore potential to contribute to containing antibiotic resistance.

## Figures and Tables

**Table 1:** Shows residual antibiotic activity of the antibiotic ciprofloxacin after photocatalytic degradation with Fe-doped ZnO nanoparticles against *Staphylococcus aureus* and *Escherichia coli*.

Test Bacteria	*Staphylococcus Aureus* (10^8^ CFU)			*Escherichia Coli* (10^8^ CFU)		
Time (min)	PCD ZOI in mm Mean ± SEM	DC ZOI in mm Mean ± SEM	PL ZOI in mm Mean ± SEM	PCD ZOI in mm Mean ± SEM	DC ZOI in mm Mean ± SEM	PL ZOI in mm Mean ± SEM
0	12 ± 0.3	12.5 ± 0.3	12.5 ± 0.2	15 ± 0.3	15 ± 0.2	14.5 ± 0.3
30	12.5 ± 0.3	12 ± 0.3	12.5 ± 0.2	14.5 ± 0.3	14.5 ± 0.2	14.5 ± 0.2
45	10 ± 0.3	8 ± 0.5	11 ± 0.2	11 ± 0.3	14 ± 0.2	14 ± 0.2
60	7.5 ± 0.2	12 ± 0.5	10 ± 0.2	12 ± 0.4	14 ± 0.2	13.5 ± 0.3
75	5.5 ± 0.2	11 ± 0.3	10.5 ± 0.2	9.5 ± 0.3	14.5 ± 0.2	12 ± 0.5
90	0	11.5 ± 0.5	9 ± 0.3	6 ± 0.2	14.5 ± 0.2	11.5 ± 0.3
105	0	9 ± 0.3	8.5 ± 0.2	0	12 ± 0.3	12 ± 0.3
120	0	10 ± 0.2	7.5 ± 0.3	0	12.5 ± 0.4	10 ± 0.3
135	0	10 ± 0.3	8 ± 0.2	0	14 ± 0.3	9 ± 0.3
150	0	10.5 ± 0.2	7 ± 0.2	0	14 ± 0.2	8.5 ± 0.2
165	0	9 ± 0.3	6 ± 0.2	0	14.5 ± 0.3	7 ± 0.2
180	0	11 ± 0.3	5.5 ± 0.2	0	14 ± 0.2	0
195	0	11 ± 0.2	0	0	14 ± 0.3	0
210	0	12 ± 0.3	0	0	13 ± 0.5	0

SEM stands for standard error of mean, calculated from the standard deviation, PCD-photocatalytic degradation, DC-dark control, PL-photolysis, *n* (number of replicates) = 6. Zone of inhibition (ZOI) = total zone (including the disc)—diameter of the disc (6 mm). The well diffusion assays were performed in accordance with the Clinical & Laboratory Standards Institute (CLSI) Guidelines. No ZOI have been observed from the solvent controls i.e., with distilled water, no contaminating bacteria were found to grow around the treated samples when poured without the test bacteria. Experimental Conditions: catalyst concentration 150 mg/L, pH 9, antibiotic concentration 10 mg/L and temperature 30 °C.
